# Kefir peptides alleviate particulate matter <4 μm (PM_4.0_)-induced pulmonary inflammation by inhibiting the NF-κB pathway using luciferase transgenic mice

**DOI:** 10.1038/s41598-019-47872-4

**Published:** 2019-08-08

**Authors:** Hsiao-Ling Chen, Kuan-Fei Hung, Chih-Ching Yen, Chun-Huei Laio, Jiun-Long Wang, Ying-Wei Lan, Kowit-Yu Chong, Hueng-Chuen Fan, Chuan-Mu Chen

**Affiliations:** 1 Department of Bioresources, Da-Yeh University, Changhua, 515 Taiwan; 2 Department of Life Sciences, and Ph.D. Program in Translational Medicine, National Chung Hsing University, Taichung, 402 Taiwan; 3 Department of Internal Medicine, China Medical University Hospital, Taichung, 404 Taiwan; 4 Division of Chest Medicine, Department of Internal Medicine, Taichung Veterans General Hospital, Taichung, 407 Taiwan; 5 Department of Medical Biotechnology and Laboratory Science, College of Medicine, Chang Gung University, Tao-Yuan, 333 Taiwan; 6 Department of Thoracic Medicine, Chang Gung Memorial Hospital at Linkou, Tao-Yuan, 333 Taiwan; 7 Department of Pediatrics, and Department of Medical Research, Tung’s Taichung Metro-harbor Hospital, Wuchi, Taichung 435 Taiwan; 8 Department of Rehabilitation, Jen-Teh Junior College of Medicine, Nursing and Management, Miaoli, 356 Taiwan; 9 The iEGG and Animal Biotechnology Center, and Rong Hsing Research Center for Translational Medicine, National Chung Hsing University, Taichung, 402 Taiwan

**Keywords:** Preventive medicine, Bioluminescence imaging, Animal biotechnology

## Abstract

Kefir peptides, generated by kefir grain fermentation of milk proteins, showed positive antioxidant effects, lowered blood pressure and modulated the immune response. In this study, kefir peptide was evaluated regarding their anti-inflammatory effects on particulate matter <4 μm (PM_4.0_)-induced lung inflammation in NF-κB-luciferase^+/+^ transgenic mice. The lungs of mice under 20 mg/kg or 10 mg/kg PM_4.0_ treatments, both increased significantly the generation of reactive oxygen species (ROS) and inflammatory cytokines; increased the protein expression levels of p-NF-κB, NLRP3, caspase-1, IL-1β, TNF-α, IL-6, IL-4 and α-SMA. Thus, we choose the 10 mg/kg of PM_4.0_ for animal trials; the mice were assigned to four treatment groups, including control group (saline treatment), PM_4.0_ + Mock group (only PM_4.0_ administration), PM_4.0_ + KL group (PM_4.0_ + 150 mg/kg low-dose kefir peptide) and PM_4.0_ + KH group (PM_4.0_ + 500 mg/kg high-dose kefir peptide). Data showed that treatment with both doses of kefir peptides decreased the PM_4.0_-induced inflammatory cell infiltration and the expression of the inflammatory mediators IL-lβ, IL-4 and TNF-α in lung tissue by inactivating NF-κB signaling. The oral administrations of kefir peptides decrease the PM_4.0_-induced lung inflammation process through the inhibition of NF-κB pathway in transgenic luciferase mice, proposing a new clinical application to particulate matter air pollution-induced pulmonary inflammation.

## Introduction

In recent years, particulate matter (PM), a major component of air pollution, has caused great concern and has been associated with a reduction in pulmonary function and exacerbation of chronic respiratory diseases such as asthma and chronic obstructive pulmonary disease (COPD)^1,2^. Air pollutants also increased the incidence of various gastrointestinal diseases and liver fibrosis and increased the morbidity and mortality of lung cancer and cardiovascular diseases, making PM a threat to human health^3,4^. Numerous hazardous components in PM are known to contain various toxins such as carbonaceous cores, polycyclic aromatic hydrocarbons (PAHs), quinones, sulfate, heavy metals, and endotoxins, which are typically accompanied by decreased visibility^5^. Based on its aerodynamic diameter, PM is crudely categorized as coarse PM, which has an aerodynamic diameter of 2.5–10 μm; fine PM, which has an aerodynamic diameter <2.5 μm; and ultrafine PM (UFPM), which has an aerodynamic diameter <0.1 μm. It’s important to highlight that the PM_10_ (aerodynamic diameter <10) include coarse, fine and UFPM. Each type of PM has a distinct composition and mediates different effects on organ health^6^. Where the effects of UFPM is mainly due to the small size of the particles, which deposit deep in the lungs, cross epithelial barriers to enter the circulation, and then impact on distal organs, leading to their entry into intracellular compartments and disruption of cell activity^7,8^. Studies showed that PM_2.5_ can bypass human innate defense mechanisms, reaching deep levels of the bronchial system and creating deposits of this PM in alveolar and terminal bronchioles^9^. However, the roles and detailed mechanisms of PM_4.0_-induced pulmonary inflammation effects still remain largely unknown. Therefore, in this study, we focus on PM_4.0_ and the development of pulmonary inflammatory process.

The inflammasome is an important component of innate immunity involved in systemic inflammation, including lung tissue’s inflammatory response. It is a multiprotein complex that is activated by damage-associated molecular patterns (DAMPs) in a two-step process: activation of NLRP3 and DAMP, and pathogen-associated molecular pattern (PAMP) induction of inflammasome assembly, including NLRP3, associated speck-like protein (ASC) and caspase-1^10,11^. The NLRP3-inflammasome complex regulates the activation of caspase-1, which catalyzes the cleavage of pro-IL-1β while the NLRP3 inflammasome activates ASC and caspase-1, which in turn leads to the maturation of the inflammatory cytokines IL-1β and IL-18^12^. Moreover, both neutrophils and macrophages are important cellular effectors of the innate immune defense, and it is clear that circulating monocytes also significantly contribute to the defense against inflammatory reactions^13^.

Kefir grains is associated with broad health benefits, since contain a complex of symbiotic components, including lactic acid, acetic bacteria, exopolysaccharide (EPS), and proteins with natural bioactive peptides with a variety of biological activities, such as antimicrobial, immunomodulatory, antiallergenic, antitumoral, antidiabetic, anti-inflammatory, antimutagenic activities and antioxidative effects^14–20^. Previous study demonstrated that administration of probiotic strain (*Lactobacillus paracasei*, one probiotics of kefir) improved PM_2.5_-induced airway hyperresponsiveness and allergic airway response, possibly through modulating Th1/Th2 immune response and IL-17 pro-inflammatory immune response in asthma mouse model^21^. In this research was established a PM_4.0_-induced lung inflammation in transgenic homozygous NF-κB-luciferase^+/+^ mice model and then was evaluated the anti-inflammatory and antioxidant effect of kefir peptide.

## Results

### Effect of PM_4.0_ exposure on pulmonary inflammation in NF-κB-luciferase^+/+^ transgenic mice

**Figure 1:**
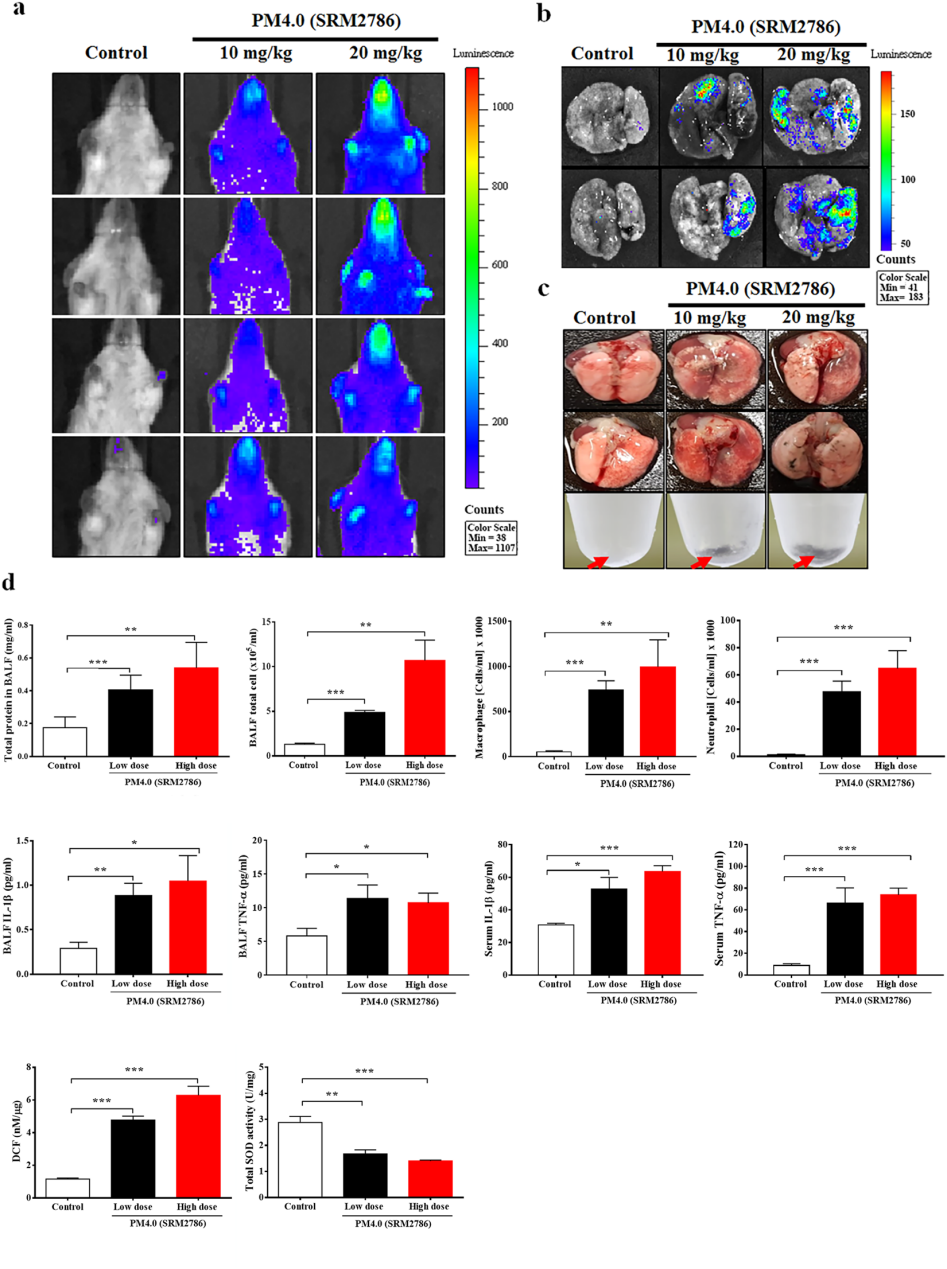
Effects of PM_4.0_ exposure on inflammation in NF-κB-luciferase^+/+^ transgenic mice. (**a**,**b**) Bioluminescence imaging of the chest cavity and lung tissue of transgenic NF-κB^+/+^ mice after exposure to saline solution, 10 and 20 mg/Kg of PM_4.0_ for 4 weeks. (**c**) Both PM_4.0_-exposed transgenic mice had more black particle deposition in the BALF and lung tissue than the control group. Red arrow: PM_4.0_ aggregation. (**d**) PM_4.0_ exposure increased the total cell and total protein levels, the macrophage and neutrophil cell counts, the inflammatory cytokine (IL-1β, TNF-α) levels, and the DCF (ROS) levels in BALF; decreased of total SOD activity in lung tissue; increased the circulating inflammatory cytokine (IL-1β, TNF-α) levels in serum compared to those in the control group. *n* = 8 per group. Data are expressed as the mean ± SD. *p < 0.05, **p < 0.01, ***p < 0.001 compared to the control group.

PM_4.0_ in both doses (10 and 20 mg/kg) increased the luminescent signals in the total chest cavity and *ex vivo* lung tissue compared to control group (without treatment) when quantified using the *In Vivo* Imaging System (IVIS) (Fig. 1a.b). Interestingly, the deposition of PM_4.0_ particles increased in the pulmonary tissue and BALF in a dose-dependent manner (Fig. 1c). PM_4.0_ in both administrations (10 and 20 mg/kg of dose) increased the total protein levels, total and relative cell counts of macrophages and neutrophils compared to those in the control group (*p* < 0.01 and *p* < 0.001) (Fig. 1d). Data showed that PM_4.0_ significantly increased the levels of IL-1β and TNF-α, in BALF and serum, compared to those in the control group (*p* < 0.05). A significant increase in the generation of extracellular ROS in the pulmonary tissue was observed in the PM_4.0_-induced groups compared with the control group (*p* < 0.001) when analyzed using DCF-DA fluorescence without difference between high and low doses of PM_4.0_ (Fig. 1d). The balance between the production of ROS and the antioxidant defense system, which includes SOD, determines the degree of oxidative stress. PM_4.0_ in both doses (10 and 20 mg/kg) decreased the total SOD activity compared to those in the control group (*p* < 0.01 and *p* < 0.001) (Fig. 1d).

### Effect of PM_4.0_ exposure on inflammatory mediator expression in NF-κB-luciferase^+/+^ transgenic mice

**Figure 2:**
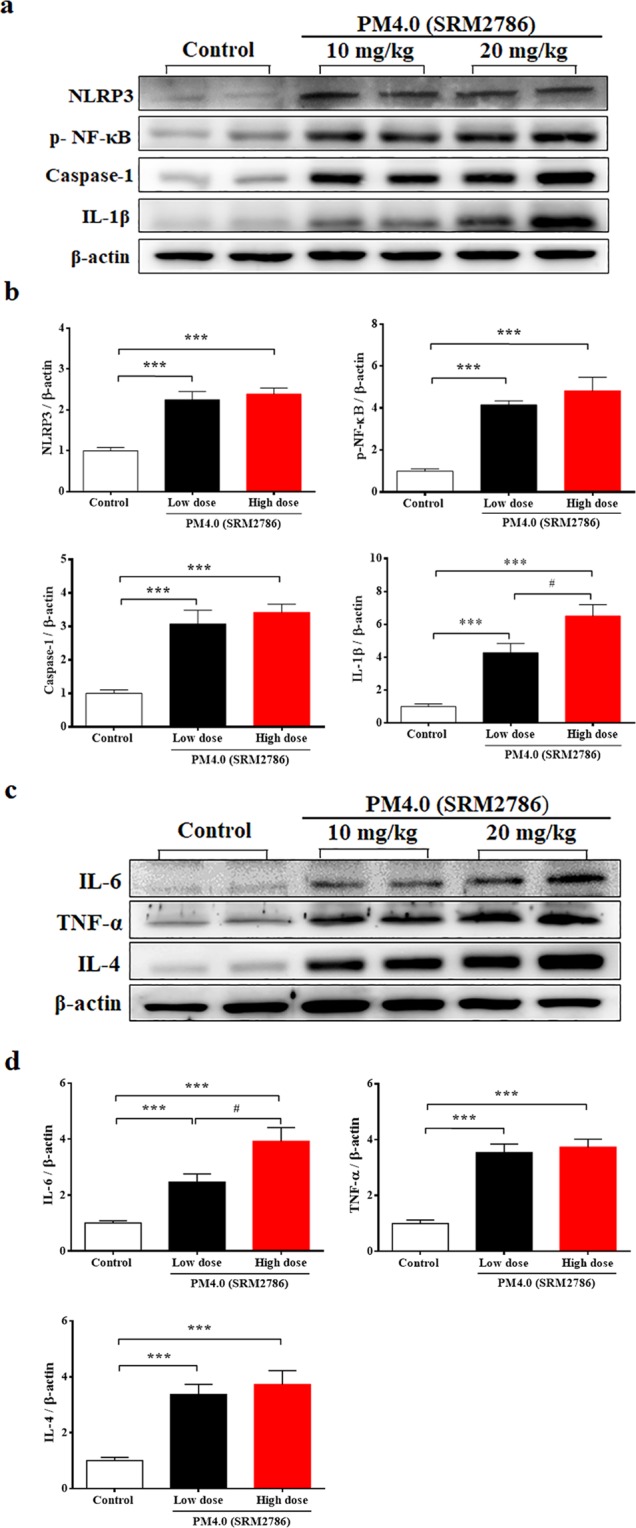
PM_4.0_ exposure increases the activation of the NLRP3-dependent and NF-κB-dependent pathways in the lung tissue of NF-κB-luciferase^+/+^ transgenic mice. (**a**) Western blot analysis of the protein expression levels of NLRP3, p-NF-κB, caspase-1 and IL-1β in different groups. (**b**) Quantification of the protein expression levels by normalization to the internal control, β-actin. (**c**) Western blot analysis of the protein expression levels of IL-6, TNF-α and IL-4 in the lung tissue of transgenic NF-κB^+/+^ mice. (**d**) Quantification of protein expression levels by normalization to the internal control, β-actin. Representative images of protein expression levels assayed by Western blotting. *n* = 8 per group. Data are expressed as the mean ± SD. *p < 0.05, ***p < 0.001 com*p*ared to the control group. ^#^*p* < 0.05 compared to the 10 mg/kg PM_4.0_-treated group.

To determine whether exposure to PM_4.0_ induced pulmonary inflammatory responses in transgenic mice, the levels of the inflammatory mediators p-NF-κB, NLRP3, caspase-1, IL-1β, TNF-α, IL-6 and IL-4 in pulmonary tissue were quantified (Fig. 2). The expression of NLRP3, p-NF-κB, caspase 1, IL-4 and TNF-α were significantly increased in the PM_4.0_ groups, without differences between them, compare to control group (*p* < 0.001). The inflammatory cytokines IL-1β and IL6 increase the expression in both group with PM_4.0_ group respect to control group (*p* < 0.001) being mayor in high dose group (*p* < 0.05) (Fig. 2).

### Effect of PM_4.0_ exposure on histopathological changes in NF-κB-luciferase^+/+^ transgenic mice

**Figure 3:**
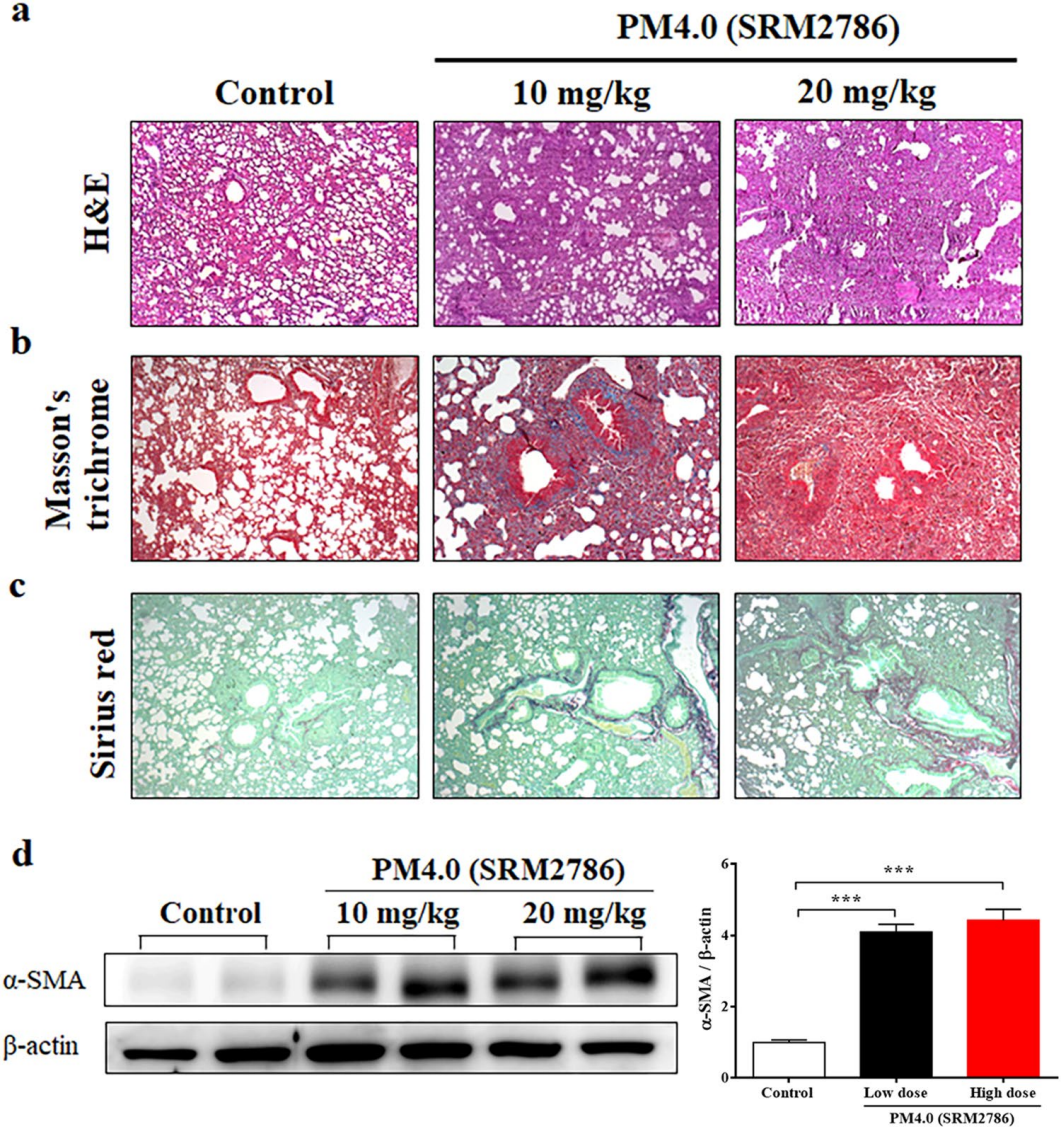
PM_4.0_ exposure increases the activation of pulmonary inflammation and fibrosis in NF-κB-luciferase^+/+^ transgenic mice. (**a**) Morphologic features of mouse lung inflammation indicated by hematoxylin and eosin (H&E) staining. The thicknesses of the bronchial wall, epithelial layer and smooth muscle in the airways of the PM_4.0_ groups were higher than those of the control group. Representative photomicrographs showing H&E staining (magnification, 100x). (**b**) Collagen deposition in the lung tissue of mice was observed by Masson’s trichrome staining. Hypertrophy, dense collagen bundles, and increased collagen deposition were present in the PM_4.0_ groups compared with the control group. Representative photomicrographs showing Masson’s trichrome staining (magnification, 100x). (**c**) Collagen fibers in the lung tissue of mice were observed by Sirius red staining. More collagen fibers were present in the PM_4.0_ groups than in the with control group. Representative photomicrographs showing Sirius red staining (magnification, 100x). The scale bars in all images are 100 μm. (**d**) Changes in the protein expression level of α-SMA in different groups normalized to the internal control, β-actin. PM_4.0_ exposure increased the α-SMA expression level in the PM4.0 groups compared with that in the control group. Representative images showing the protein expression levels assayed by Western blotting. *n* = 8 per group. Data are expressed as the mean ± SD. ***p < 0.001 compared to the control group.

Lung histopathology was examined and showed pulmonary edema and alveolar infiltration of neutrophils in the PM_4.0_ groups (Fig. 3a). After PM_4.0_ administrations (10 and 20 mg/kg of dose), the amount of collagen was significantly increased compared to the control group (Fig. 3b,c). Furthermore, analysis of α-smooth muscle actin (α-SMA) in lung tissues by Western blotting also showed significant increases in the groups exposed to low and high doses of PM_4.0_ compared to the control group, without difference between them (*p* < 0.001). The results suggested that the lung inflammation and fibrosis in the group exposed to the low dose (10 mg/kg) of PM_4.0_ daily were sufficient, so we choose the low dose of PM_4.0_ to further evaluate the pulmonary inflammation status after kefir peptides treatment in a preventive animal trial.

### Effect of kefir peptides on PM_4.0_-induced NF-κB activation in NF-κB-luciferase^+/+^ transgenic mice

**Figure 4:**
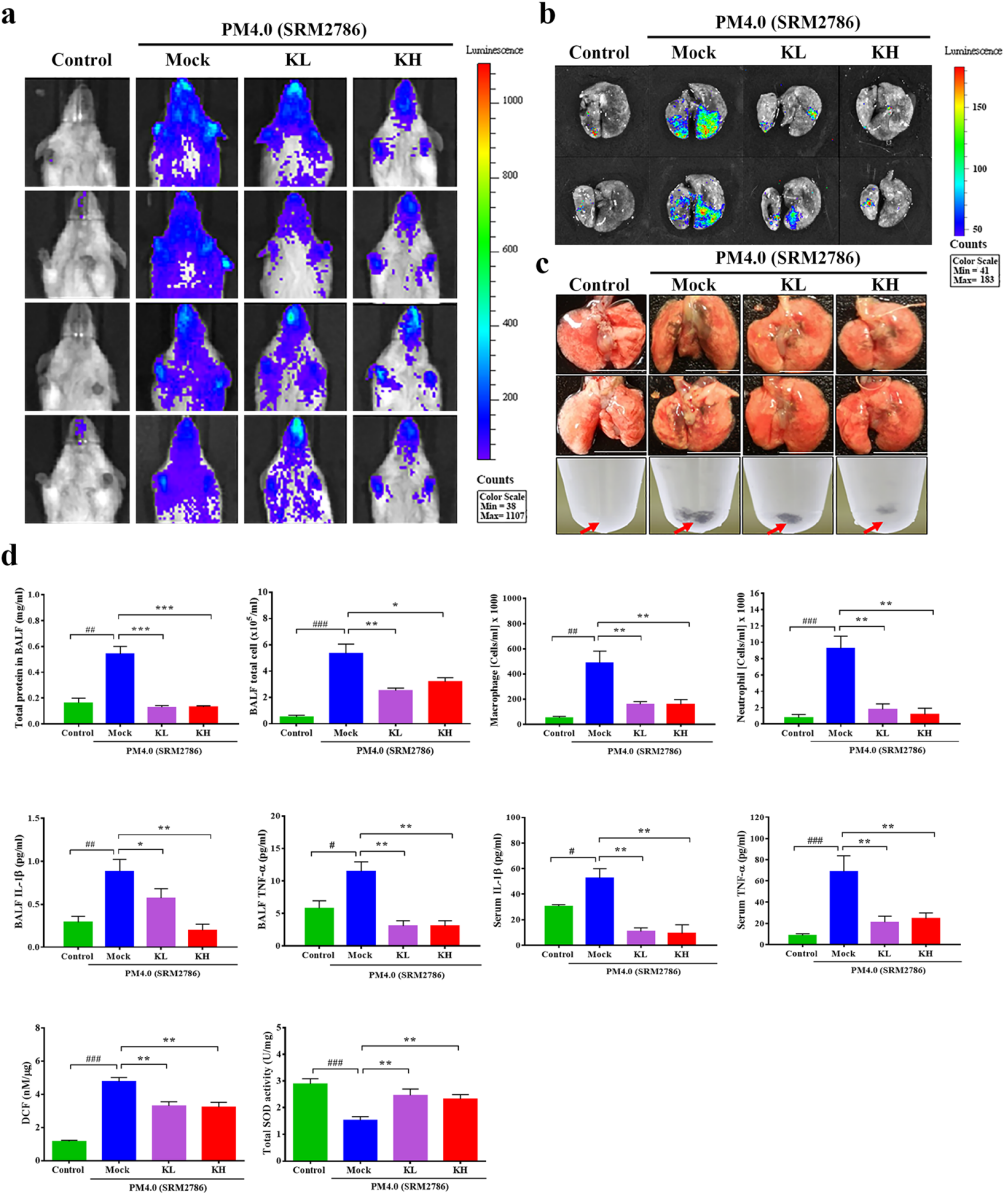
Kefir peptides decrease the PM_4.0_-induced inflammation in NF-κB-luciferase^+/+^ transgenic mice. (**a**,**b**) Kefir peptides suppressed the PM_4.0_-induced signal observed via bioluminescence imaging of the chest cavity and lung tissue in mice compared with that in the PM_4.0_/Mock group. (**c**) Kefir peptides reduced the PM_4.0_-induced black particle deposition in the BALF and lung tissue compared with that in the PM_4.0_/Mock group. Red arrow: PM_4.0_ aggregation. (**d**) Kefir peptides decreased the PM_4.0_-induced changes in total cell number, total protein level, macrophage and neutrophil cell counts, inflammatory cytokine (IL-1β, TNF-α) level, and ROS level in BALF; increased the SOD activity in lung tissue; and decreased the circulating inflammatory cytokine (IL-1β, TNF-α) levels in serum compared those in the PM_4.0_/Mock group. *n* = 8 per group. Data are expressed as the mean ± SD. ^#^*p* < 0.05, ^##^*p* < 0.01, ^###^*p* < 0.001 compared to the control group. *p < 0.05, ** *p* < 0.01, ***p < 0.001 compared to PM_4.0_/Mock group.

PM_4.0_ stimulated the luminescence signal in the chest and lung tissue, but the luciferase signals in the PM_4.0_ + KL and PM_4.0_ + KH groups were significantly lower than that in the PM_4.0_ + Mock group (Fig. 4a,b). The deposition of PM_4.0_ particles was significantly increased in the pulmonary tissue and BALF in the PM_4.0_ + Mock group; however, treatments with kefir peptides significantly decreased the PM_4.0_ deposition compared to that in the PM_4.0_ + Mock group (Fig. 4c).

### Effect of kefir peptides on PM_4.0_-induced pulmonary inflammation and oxidative status in NF-κB-luciferase^+/+^ transgenic mice

The generation of total proteins, total cells, inflammatory cells (neutrophils and macrophages), inflammatory cytokines (IL-1β and TNF-α) and extracellular ROS in BALF, as well as the inflammatory cytokines in serum, were significantly higher in the PM_4.0_ + Mock group than in the control group (*p* < 0.01). However, treatments with kefir peptides (KH and KL dose) led to a significant decrease in ROS, inflammatory cells and cytokines compared to those in the PM_4.0_ + Mock group (*p* < 0.01) (Fig. 4d). In addition, the total SOD activity in pulmonary tissue were significantly lower in the PM_4.0_ + Mock group than in the control group (*p* < 0.001), and treatments with kefir peptides significantly increased the SOD activity compared to that in the PM_4.0_ + Mock group (*p* < 0.01), without differences between them (Fig. 4d).

### Effect of kefir peptides on inflammatory mediator expression in PM_4.0_-treated NF-κB-luciferase^+/+^ transgenic mice

**Figure 5:**
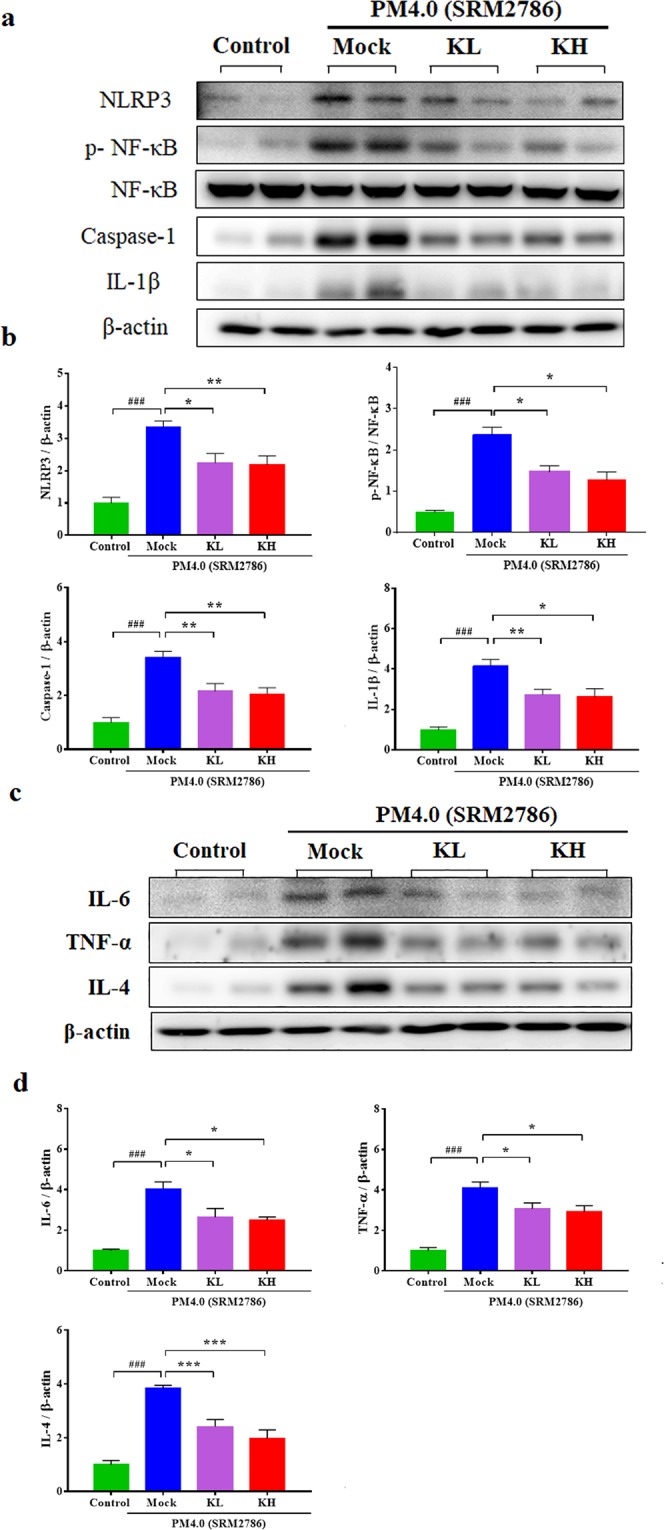
Kefir peptides mitigate the PM_4.0_-induced activation of the NLRP3-dependent and NF-κB-dependent pathways in the lung tissue of NF-κB-luciferase^+/+^ transgenic mice. (**a**) Kefir peptides reduced the PM_4.0_-induced protein expression levels of NLRP3, p-NF-κB, NF-κB, caspase-1 and IL-1β compared with those in the PM_4.0_/Mock group. (**b**) Quantification of the protein expression levels by normalization to the internal control, β-actin, while the p-NF-κB expression was normalized by NF-κB. (**c**) Kefir peptides reduced the PM_4.0_-induced protein expression levels of IL-6, TNF-α and IL-4 compared with those in the PM_4.0_/Mock group. (**d**) Quantification of the protein expression levels by normalization to the internal control, β-actin. Representative images showing the protein expression levels assayed by Western blotting. *n* = 8 per group. Data are expressed as the mean ± SD. ^###^*p* < 0.001 compared to the control group. *p < 0.05, **p < 0.01, ***p < 0.001 compared to the Mock group.

The ratio of p-NF-κB/NF-κB protein expression was significantly increased in the PM_4.0_ + Mock group compared to that in the control group (*p* < 0.001), and treatments with kefir peptides significantly decreased the p-NF-κB level and p-NF-κB/NF-κB ratio compared to that of the PM_4.0_ + Mock group (Fig. 5a,b). In this study, we observed that treatments with either low dose or high dose of kefir peptides could reduce NF-κB expression and thus subsequently decrease NLRP3, caspase-1, IL-1β, IL-6, TNF-α and IL-4 expression (Fig. 5a–d).

### Effect of kefir peptides on histopathological changes in the lungs of NF-κB-luciferase^+/+^ transgenic mice

**Figure 6:**
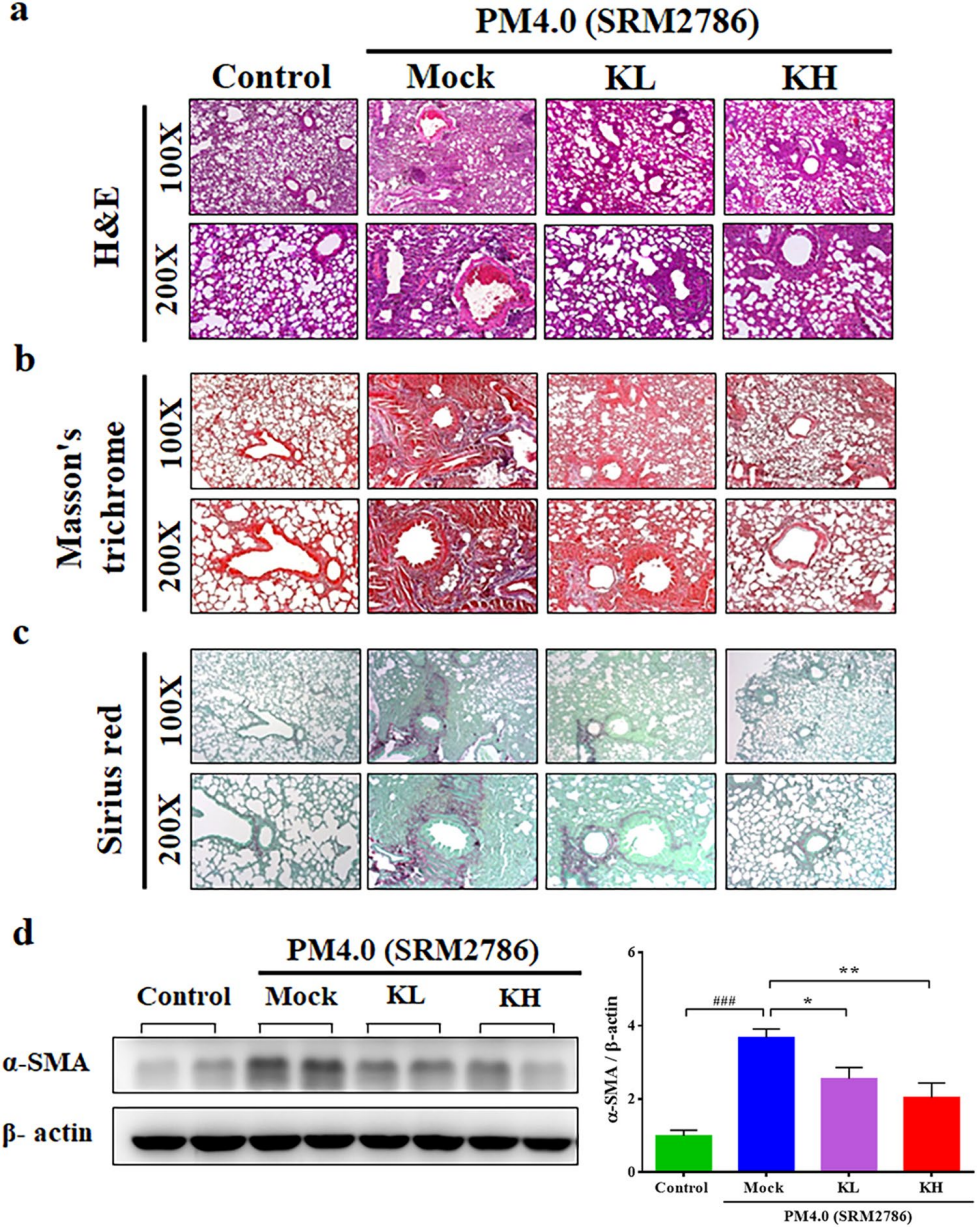
Kefir peptides improved the PM_4.0_-induced pulmonary inflammation and fibrosis in NF-κB-luciferase^+/+^ transgenic mice. (**a**) Morphologic features of the mice lung inflammation observed by H&E staining. Kefir peptides reduced the PM_4.0_-induced pulmonary inflammation in mice compared with that in the PM_4.0_/Mock group. (**b**) The collagen deposition in the lung tissue of the mice was observed by Masson’s trichrome staining. Kefir peptides reduced the PM_4.0_-induced collagen deposition in the pulmonary tissue of the mice compared that in the PM_4.0_/Mock group. (**c**) Collagen fibers in the lung tissue of the mice were observed by Sirius red staining. Kefir peptides reduced the PM_4.0_-induced collagen fibers in the pulmonary tissue of mice compared with that in the PM_4.0_/Mock group. The scale bars in all images indicate 100 μm. Lower magnification (100x) images of lung tissues are shown in the upper panel. Higher magnification (200x) images are shown in the lower panel. (**d**) Changes in the protein expression level of α-SMA in different groups normalized to an internal control, β-actin. Kefir peptides reduced the PM_4.0_-induced protein expression levels of α-SMA compared that in the PM_4.0_/Mock group; the levels were normalized to the internal control, β-actin. Representative images of the protein expression levels assayed by Western blotting. *n* = 8 per group. Data are expressed as the mean ± SD. ^###^*p* < 0.001 compared to the control group. *p < 0.05, **p < 0.01, ***p < 0.001 compared to the PM_4.0_/Mock group.

To further confirm the protective effect of kefir peptides on PM_4.0_-induced lung inflammation, a histopathological examination of the lungs was performed after 4 weeks of PM_4.0_ exposure. Pulmonary edema, alveolar infiltration of neutrophils and lung fibrosis were evident in the PM_4.0_ + Mock group (Fig. 6a–c). However, the groups treated with either low-dose (KL) or high-dose (KH) kefir peptides exhibited lower amounts of neutrophil infiltration, lung edema and lung fibrosis, including collagen deposition and collagen fibers (Fig. 6b,c). In addition, the expression of α-SMA protein was significantly higher in the PM_4.0_ + Mock group than in the control group (*p* < 0.001), and treatments with kefir peptides at either the low dose or the high dose significantly decreased the α-SMA level compared to that of the PM_4.0_ alone/Mock group (*p* < 0.01) (Fig. 6d).

## Discussion

In the present study, we note three major findings indicating that kefir peptides alleviate PM_4.0_-induced pulmonary inflammation in NF-κB-luciferase^+/+^ transgenic mice through NF-κB pathway inhibition. First, exposure to PM_4.0_ through intratracheal instillation once a day for 4 weeks successfully induces pulmonary inflammation in transgenic mice. PM_4.0_ exposure induces inflammatory cell infiltration, oxidative stress and inflammatory mediator overexpression in lung tissue by activating the NF-κB pathway. Second, kefir peptides reduce the ROS levels; decrease NF-κB activation, proinflammatory cytokine production and inflammatory cell infiltrates; and increase the total SOD activity in lungs. Third, the antioxidant effect and the subsequent reduction in activation of the NF-κB, NLRP3-dependent inflammasome and caspase-1 pathways contribute to the complex molecular anti-inflammatory mechanisms of kefir peptides.

The medical imaging system using NF-κB-luciferase^+/+^ transgenic mice carrying the luciferase gene driven by the NF-κB promoter is a potential animal model for monitoring inflammation and the effects of treatments. The best advantage is that this method provides noninvasive, real-time and whole-body screening. The results from the medical imaging agree with our expectations to some extent; the most evident organ expressing luciferase *in vivo* was the lung. The present study demonstrated, for the first time, the anti-inflammatory effect of kefir peptides on PM_4.0_-induced lung inflammation in NF-κB-luciferase^+/+^ transgenic mice, as shown in bioluminescence images obtained by the *In Vivo* Imaging System (IVIS)^22,23^. The imaging results show us that the lung is one of the organs with oxidative stress and inflammatory responses after exposure to PM_4.0_ (Fig. 1a,b).

**Figure 7:**
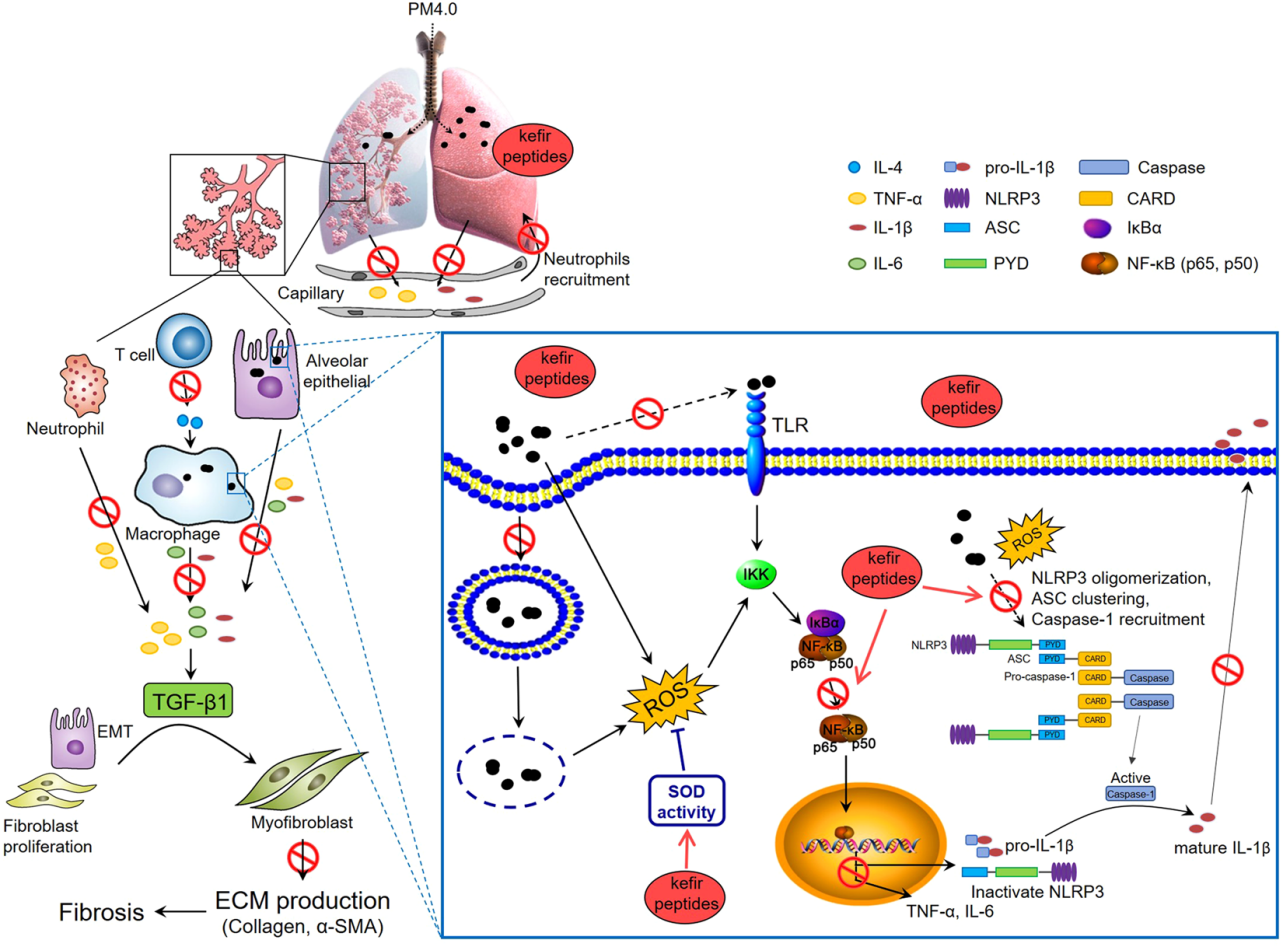
Hypothetical scheme of the kefir peptides regulatory pathway. The effects of kefir peptides on the anti-inflammatory response are hypothesized to occur through the NF-κB signaling pathway. The diagram shows that kefir peptides might inhibit the expression of p-NF-κB, NLRP3, caspase-1, IL-1β, IL-6, TNF-α, α-SMA, and IL-4. Kefir peptides might also increase the SOD activity to reduce the ROS generation.

PM_2.5_ is well known to induce prooxidant and proinflammatory actions^9,24,25^, but the PM_4.0_-induced effects on inflammatory responses in mice were not known. Previous reports demonstrated that PM_2.5_ could be internalized into cells through endocytosis processes and have potentials to activate NLRP3 inflammasome through activation of NF-κB-dependent cascade and assembly of inflammasome complex (including cathepsin B release, ROS production and potassium efflux), as a result of pulmonary fibrosis^26,27^. To our knowledge, this is the first report showing that PM_4.0_ exposure leads to inflammatory responses in the lung and the occurrence of systematic inflammation, resulting in the release of inflammatory cytokines, which can induce lung inflammation through mechanisms that are similar to those for PM_2.5_^28–30^. In this study, PM_4.0_ activated p-NF-κB, leading to activation of the NLRP3 inflammasome, which induced caspase-1 activation and thus the production of proinflammatory IL-1β. The elevated IL-1β simultaneously activated the expression of TNF-α, IL-6 and IL-4 (Fig. 2), which play a crucial role in the inflammatory pathway^31–33^. Nevertheless, treatments with kefir peptides significantly decreased the protein expression of p-NF-κB, NLRP3, caspase-1, IL-1β, TNF-α, IL-6 and IL-4 and increased the SOD activity in NF-κB-luciferase^+/+^ transgenic mice (Figs 4 and 5). Taken together, these results indicate that kefir peptides affects p-NF-κB, NLRP3, caspase-1, IL-1β, TNF-α, IL-6 and IL-4, all of which reduce the inflammatory response, by inactivating NF-κB signaling (luciferase expression, phosphorylated NF-κB, NLRP3-dependent inflammasome and caspase-1). A hypothetical scheme of the kefir peptides regulatory pathway against PM_4.0_-induced lung inflammation is shown in Fig. 7.

The lungs are susceptible to damage by airborne particles, as observed in the histological sections, BALF and serum samples. Following PM exposure, inflammatory responses are stimulated and numerous inflammatory cytokines are released from the lung parenchyma^34,35^. Several pulmonary diseases including asthma, acute lung injury, COPD and acute respiratory distress syndrome (ARDS), are associated with abnormal TNF-α, IL-1β and IL-6 expression^36^. To investigate whether the protective effect of kefir peptides against PM_4.0_-induced pulmonary injury was associated with inflammation, TNF-α and IL-1β levels were measured. The present study revealed that kefir peptides treatment significantly decreased TNF-α and IL-1β protein levels in BALF and serum. These results indicate that kefir peptides treatment can ameliorate PM_4.0_-induced damage by suppressing inflammation. In addition, pulmonary inflammation and fibrosis, including inflammatory cell infiltration, interstitial edema, collagen deposition, collagen fibers and overexpression of α-SMA protein (Fig. 3), were observed in PM_4.0_-treated mice, suggesting that the pulmonary biofilm and parenchymal cells were damaged. This damage was significantly attenuated by kefir peptides treatment (Fig. 6). These results indicate that kefir peptides could exert protective effects against PM_4.0_-induced pulmonary inflammation.

Kefir, which originated in the North Caucasian mountains, is rich in protein complex, EPS and peptides^15,16^. Recent research showed that kefir products comprise many of the bacterial strains that may survive in the digestive process and actually reach the gut, that results in transient changes in the inflammatory cytokines and achieve long-term benefits through regulating the gut barrier and microbiota, in both *in vivo* and *in vitro* experiments^37^. In addition, analysis of the peptides in bovine kefir revealed 236 casein-derived unique peptides in kefir grains, including 16 peptides with angiotensin-converting enzyme-inhibitory, antimicrobial, immunomodulating, opioid, mineral-binding, antioxidant, and antithrombotic effects^38^.

Our previous *in vivo* animal study demonstrated that kefir peptides improve hyperlipidemia and obesity via inhibition of lipogenesis, modulation of oxidative damage, and stimulation of lipid oxidation in high‐fat‐diet‐induced obese rats^17^. The mechanisms of kefir probiotic products to exhibit health benefits is through modulating the gut immune system. Many studies have proved that kefir involved in modulating the inflammatory responses possibly through regulating NF-κB signaling pathway in both of intestinal epithelial cells (*in vitro*)^39^ and LPS-induced acute kidney injury mouse (*in vivo*)^40^. Lee *et al*.^41^ mentioned that *Lactobacillus acidophilus* (main probiotics of kefir) modulates inflammatory activity by decreasing the levels of toll-like receptor-4 (TLR4)-induced NF-κB activity in peripheral blood mononuclear cells of LPS-challenged porcine model. Kefir regulates Th1-to-Th2 shift of immune responses and others mentioned that kefir increases in some pro-inflammatory cytokines such as TNF-α, IFN-γ, or IL-12 as an initial reaction of the immune system to TLR agonists present, which resulted in attenuating following further interaction with the immune cells^42,43^. Kefir peptides improved nonalcoholic fatty liver diseases via activation of Janus kinase 2 (JAK2) signal transduction through the JAK2/signal transducer and activator of transcription protein 3 (STAT3) and JAK2/AMP-activated protein kinase (AMPK) pathways in a high fructose-induced fatty liver animal model^44^. Kefir significantly improved the body weight, energy expenditure and basal metabolic rate in nonalcoholic fatty liver disease by inhibiting the lipogenesis pathway in leptin receptor-deficient *ob/ob* mice^45^. In addition, one study demonstrated that polysaccharides of *Astragalus* and *Codonopsis pilosula* improved the alveolar macrophage phagocytosis and inflammation in COPD mice exposed to PM_2.5_^46^. Collectively, the present study demonstrated, for the first time, that treatment with 150 or 500 mg/kg body weight of kefir peptides in NF-κB-luciferase^+/+^ transgenic mice could be protecting against the lung inflammation and oxidative stress caused by PM_4.0_ exposure.

## Conclusion

In summary, our results demonstrate that PM_4.0_-induced inflammatory cell infiltration, oxidative stress and overexpression of inflammatory mediators in lung tissue by activating the NF-κB pathway in NF-κB-luciferase^+/+^ transgenic mice. However, treatment with kefir peptides reduced the PM_4.0_-induced generation of ROS, suppressed p-NF-κB, NLRP3, caspase-1, IL-1β, TNF-α, IL-6, IL-4 and α-SMA expression and increased the SOD activity. Therefore, kefir peptides alleviated PM_4.0_-induced lung inflammation through inhibition of NF-κB signaling and may have the potential for clinical applications involving particulate matter air pollution.

## Methods

### PM_4.0_ (SRM 2786) characterization

PM_4.0_, standard reference material (SRM) No. 2786, is a fine atmospheric particulate matter with a mean particle diameter <4 μm; PM_4.0_ was purchased from the European Virtual Institute for Speciation Analysis (EVISA, Gaithersburg, MD, USA). SRM 2786 is an analytical method for the determination of selected polycyclic aromatic hydrocarbons (PAHs), nitro-substituted PAHs (nitro-PAHs), polybrominated diphenyl ether (PBDE) congeners, hexabromocyclododecane (HBCD) isomers, sugars, polychlorinated dibenzo-*p*-dioxin (PCDD) and dibenzofuran (PCDF) congeners, inorganic constituents, and particle-size characteristics in atmospheric particulate material and similar matrices^47–49^. Detailed information about the PAHs, trace elements and inorganic constituents of PM_4.0_ can be found in Supplementary Tables S1, S2 and S3, respectively.

### Kefir peptide obtaining

Kefir peptides powder was purchased from Phermpep Co. (Taichung, Taiwan) and was produced via kefir grain fermentation at 20 °C for 20 h in sterilized milk. The grains were passed through a sieve and reinoculated (10%, wt/vol) into sterilized fresh milk, and the incubation was performed according to the previously described preparation methods^17,18,45^. After the grains were filtered, the fermented products were spray-dried into kefir peptides powder using a spray dryer. The peptide content was determined according to the OPA (O-phthaladehyde) method, using triglycine as the standard. The sample or standard solution (5 μL) was mixed with 200 μL of OPA reagent (50 mM borax, 1% SDS, 0.5% thiolactic acid and 1.25 mg/mL OPA). After 2 min of incubation at room temperature, the absorbance was measured at 340 nm. The total peptide content was expressed as triglycine equivalents in g per 100 g sample. The peptide content in the kefir peptides powder (Phermpep Co.) was 23.1 g/100 g.

### Animal and experimental model

NF-κB-luciferase^+/+^ transgenic mice carry the luciferase gene driven by the NF-κB promoter; thus, the luciferase activity reflects the NF-κB activity^22,23^. Female homozygous transgenic mice of 8 weeks old were given a standard laboratory diet and distilled water *ad libitum* and were kept on a 12-h light/dark cycle at 24 ± 2 °C. These mice were randomly assigned in three groups (n = 8): the first group without treatment (control group), second group (10 mg/kg of PM_4.0_) and the last group (20 mg/kg of PM_4.0_). PM_4.0_-induced lung inflammation was established via intratracheal instillation once a day for 4 weeks. Additionally, because the preliminary results did not showed differences between the low and high dose of PM_4.0_, the group with low dose was chosen for the treatment with kefir peptides. Therefore, homozygous transgenic mice were randomly assigned to four groups for treatment (*n* = 8): (1) a normal control group receiving no treatment (Control group), as a negative control; (2) a group treated with 10 mg/kg PM_4.0_ alone (PM_4.0_ + Mock group); (3) a group treated with 10 mg/kg PM_4.0_ plus 150 mg/kg low-dose kefir peptides (PM_4.0_ + KL group); and (4) a group treated with 10 mg/kg PM_4.0_ plus 500 mg/kg high-dose kefir peptides (PM_4.0_ + KH group)^50^. Two groups were fed kefir peptides one hour before the intratracheal administration of PM_4.0_ (daily, 4 weeks). Mice were sacrificed at 12 weeks of age after 4 weeks of kefir peptides treatment. At the end of the experiment, each mouse was anesthetized, and pulmonary tissues were collected for bronchoalveolar lavage fluid (BALF), pathological histology, and protein extraction as described previously^51,52^. All animal experiments were performed according to the guidelines and were approved by the Institutional Animal Care and Utilization Committee of National Chung Hsing University, Taiwan (IACUC No. 104-077R).

### Bioluminescence imaging

Imaging was performed with the IVIS Imaging System 200 Series (Xenogen Corp., Alameda, CA, USA) with the camera set at the highest sensitivity. NF-κB-luciferase^+/+^ transgenic mice were injected intraperitoneally with luciferin (Promega, Los Altos, CA, USA) at 150 mg/kg in a volume of 200 μL and anesthetized with isoflurane^53^. After 5 min, the mice were placed supine in the chamber and imaged for 90 sec by the IVIS Imaging System. Photons were quantified using Living Image^®^ software (Xenogen Corp., Alameda, CA, USA) and the intensity of the signal was expressed as photons/sec/cm^2^.

### Histological analysis

Pulmonary tissue was fixed with 10% formalin (Macron Fine Chemicals™, Avantor Performance Materials, Center Valley, PA, USA) and embedded in paraffin wax. Paraffin-embedded sections were examined using hematoxylin and eosin (H&E), Masson’s trichrome and picrosirius red staining as previously described^51,54,55^. The severity of collagen deposition and lung fibrosis was assessed by measuring the Masson’s trichrome and picrosirius red staining, respectively^56,57^.

### Western blot analysis

Expression of pulmonary tissue protein was measured by Western blotting as previously described^51^. Briefly, pulmonary tissues were homogenized in 500 μL of radioimmunoprecipitation assay (RIPA) buffer (EMD Millipore, Billerica, MA, USA). The homogenates were centrifuged at 12,000 rpm for 30 min at 4 °C. The protein (50 μg) was then separated by SDS-PAGE in a 10% polyacrylamide gel and electrotransferred onto a polyvinylidene difluoride membrane. The membranes were incubated in blocking solution (5% BSA) at room temperature for 1 h. The membranes were washed three times (5 min each) with 0.1% T-TBS and then incubated with primary antibody (NLRP3, NF-κB, p-NF-κB, caspase-1, IL-1β, IL-6, TNF-α, IL-4, α-SMA and β-actin; Cell Signaling Technology, Inc., Danvers, MA, USA) in 0.05% T-TBS containing 2.5% BSA at room temperature for 2 h. After washing, the membranes were incubated with peroxidase-conjugated anti-mouse/rabbit antibody (Abcam, Inc., Cambridge, MA, USA) in 0.01% T-TBS at room temperature for 1 h. The membranes were developed with an enhanced chemiluminescence (ECL, Millipore Corporation, Billerica, MA, USA) detection system.

### Superoxide dismutase (SOD) activity in lung extracts

Pulmonary tissues were homogenized in 300 μL of RIPA buffer. The homogenates were centrifuged at 12,000 rpm for 30 min at 4 °C. To quantify total SOD activity, a water-soluble tetrazolium monosodium salt (WST-1) assay (SOD Assay Kit-WST; Dojindo Molecular Technologies, Inc., Rockville, MD, USA) was performed in a 96-well plate, with bovine erythrocyte SOD1 as a standard. Aliquots of the solution were immediately pipetted into 96-well flat-bottom microtiter plates containing three empty blanks, a range of concentrations of the SOD standard, and a range of concentrations of each lung extract. The rates of WST-1 reduction were measured via the OD_450_ value using a microplate reader (Thermo Scientific, Waltham, MA, USA). All determinations of SOD activity were made in triplicate^58^.

### Bronchoalveolar lavage fluid (BALF)

The trachea was exposed with a midline incision and cannulated with a modified 21-gauge needle. After euthanization, the BALF was flushed 3 times with 500 µL of sterile endotoxin-free saline each time. An average of 80% BALF was recovered after each lavage. The BALF was combined and centrifuged at 500 rpm for 10 min at 4 °C. The cell pellets were resuspended in 1 mL of PBS, and cell counts were performed^59^. The total number of cells in BALF was determined by staining with Liu’s stain to count the different cell types by using a hemocytometer. The supernatant was subjected to total protein analysis using a bicinchoninic acid (BCA) protein assay (Pierce, Rockford, IL, USA).

### Measurement of reactive oxygen species (ROS) generation

The generation of ROS, including hydrogen peroxide (H_2_O_2_), hydroxyl radicals (^•^OH) and peroxynitrite (ONOO^−^/ONOOH), in the perfused lungs was monitored via 2′, 7′-dichlorodihydrofluorescein diacetate (H_2_DCF-DA) fluorescent probe (*In Vitro* ROS/RNS Assay Kit; Cell Biolabs, Inc., San Diego, CA, USA) as previously described^60^. After internalization, the acetate group of the nonfluorescent molecule is cleaved by intracellular esterases to form H_2_DCF, which serves as a substrate for intracellular ROS to generate the highly fluorescent DCF. Fluorescence was measured with a spectrofluorometer at 480 nm excitation and 530 nm emission wavelengths. Data are expressed in relative fluorescence units for each cell.

### Measurement of cytokine levels

Blood samples were clotted at 4 °C for 60 min and then centrifuged for 10 min at 10,000 rpm. The serum levels of IL-1β and TNF-α were measured in the overnight fasting serum and assayed using commercially quantitative enzyme-linked immunosorbent assay (ELISA) kits (Abcam Inc., Cambridge, MA, USA) according to the manufacturer’s instructions.

### Statistical analysis

The data are presented as the means ± standard error of the mean (SEM). All statistical analyses were performed by using Statistical Package for the Social Sciences (SPSS) statistical software for Windows version 20.0 (SPSS Inc., Chicago, IL, USA) and two-way ANOVA with Duncan’s test. *P* < 0.05 was considered to indicate a statistically significant difference.

### Ethics approval and consent to participate

All animal experiments were performed according to the guidelines and were approved by the Institutional Animal Care and Utilization Committee of National Chung Hsing University, Taiwan (IACUC No. 104-077 R).

## Supplementary information

Supplementary Tables

## Data Availability

All data and materials are included in the article and its Supplementary Information files.
