# Supplementary material for: Kefir peptides alleviate particulate matter <4 μm (PM4.0)-induced pulmonary inflammation by inhibiting the NF-κB pathway using luciferase transgenic mice
Source: Sci Rep. 2019 Aug 8;9:11529. doi: 10.1038/s41598-019-47872-4 (PMC6687726; doi:10.1038/s41598-019-47872-4)
Supplement: Supplementary file 1 — Supplementary Tables [file 41598_2019_47872_MOESM1_ESM.pdf]

Scientific Reports

Supplementary Information

Kefir peptides alleviate particulate matter < 4 µm ($PM_{4.0}$)-induced pulmonary inflammation by inhibiting the NF-κB pathway using luciferase transgenic mice

Hsiao-Ling Chen[1†], Kuan-Fei Hung[2†], Chih-Ching Yen[2,3†], Chun-Huei Laio[2], Jiun-Long Wang[2,4], Ying-Wei Lan[2,5], Kowit-Yu Chong[5,6], Hueng-Chuen Fan[7,8*], and Chuan-Mu Chen[2,9*]

***Corresponding authors**: **Chuan-Mu Chen, Ph.D.**

Professor/Dean

Department of Life Sciences,

College of Life Sciences,

National Chung Hsing University

No. 250, Kuo Kuang Rd., Taichung 402, Taiwan

Phone: 886-4-2285-6309; Fax: 886-4-2287-4740

E-mail: chchen1@dragon.nchu.edu.tw

Hueng-Chuen Fan, MD., Ph.D.

Professor and Director

Department of Pediatrics, Tungs' Taichung Metroharbor Hospital, No. 699, Sec. 8, Taiwan Blvd., Wuchi, Taichung 435, Taiwan

Phone: 886-4-2658-1919; Fax: 886-4-2658-2193

E-mail: fanhuengchuen@yahoo.com.tw

Table S1. Certified mass fraction values for selected PAHs in $PM_{4.0}$ (SRM2786)

Selected PAHs	Mass Fraction[a] (mg/kg)
Fluorene[b,c,d,e,f]	0.195 ± 0.014
Fluoranthene[b,c,d,e,f,g]	10.28 ± 0.36
Pyrene[b,c,d,e,f,g]	8.01 ± 0.22
Benzo[*ghi*]fluoranthene[b,c,d,e,f,,g]	3.158 ± 0.098
Benzo[*c*]phenanthrene[b,c,d,e,f,g]	1.597 ± 0.052
Benz[*a*]anthracene[c,d,e,f]	4.82 ± 0.17
Chrysene[c,d]	6.82 ± 0.53
Triphenylene[c,d,g]	1.794 ± 0.041
Benzo[*b*]fluoranthene[b,e,f,g]	7.51 ± 0.36
Benzo[*j*]fluoranthene[b,e,f]	4.37 ± 0.32
Benzo[*k*]fluoranthene[b,c,d,e,f]	3.48 ± 0.32
Benzo[*a*]fluoranthene[b,c,d,e,f]	0.898 ± 0.037
Benzo[*e*]pyrene[b,c,d,e,f,g]	4.77 ± 0.28
Benzo[*a*]pyrene[b,c,d,e,f,g]	3.70 ± 0.13
Perylene[b,c,d,e,f,g]	0.769 ± 0.020
Benzo[*ghi*]perylene[b,c,d,e,f,g]	5.60 ± 0.41
Indeno[1,2,3-*cd*]pyrene[b,c,d,e,f,g]	4.87 ± 0.36
Dibenz[*a,c*]anthracene[b,e,f,g]	0.509 ± 0.052
Dibenz[*a,j*]anthracene[b,c,d,e,f,g]	0.610 ± 0.015
Dibenz[*a,h*]anthracene[b,e,f,g]	0.717 ± 0.029
Benzo[*b*]chrysene[b,c,d,e,f,g]	0.662 ± 0.022
Picene[b,c,d,e,f,g]	1.242 ± 0.031
Coronene[b,c,d,e,f]	2.156 ± 0.087
Dibenzo[*b,k*]fluoranthene[b,c,d,e,f]	1.013 ± 0.082
Dibenzo[*a,e*]pyrene[b,c,d,e]	0.812 ± 0.081

[a] The certified value is a weighted mean of the results from three to six analytical methods[S1]. The uncertainty listed with each value is an expanded uncertainty about the mean, with the coverage factor, *k* = 2 (approximately 95% confidence interval), calculated by combining a pooled within-method variance with a between-method variance following the ISO/JCGM Guide.

[b] GC/EI-MS Ia using PFE at 150 °C with toluene followed by analysis on a DB-17 column.

[c] GC/EI-MS IVa using PFE at 100 °C with toluene followed by analysis on a DB-XLB column.

[d] GC/EI-MS IVb using PFE at 150 °C with toluene followed by analysis on a DB-XLB column.

[e] GC/EI-MS V using PFE at 100 °C with DCM followed by analysis on a DB-17 MS column.

[f] GC/EI-MS II using PFE at 100 °C with DCM followed by analysis on a DB-17 MS column.

[g] GC/EI-MS Ib using PFE at 150 °C with toluene followed by analysis on a LC-50 column.

Table S2. Certified mass fraction values for selected trace elements in $PM_{4.0}$ (SRM2786)

Trace elements	Mass Fraction[(a)] (mg/kg)
Mercury[(a)]	5.32 ± 0.14
Aluminum[(b)]	33480 ± 700
Cadmium[(a)]	4.34 ± 0.07
Chromium[(b)]	462.2 ± 1.5
Copper[(b)]	847 ± 13
Lead[(a)]	286 ± 3
Manganese[(b)]	780 ± 39
Vanadium[(b)]	85.5 ± 6.5

[(a)] The certified mass fraction value is the mean of results obtained using one definitive analytical technique (ID-ICP-MS). The expanded uncertainty, *U*, is calculated as *U* = *ku*c, where *u*c is the standard error of the dry-mass corrected analyte mean, and the coverage factor, *k*, is determined from Student's *t*-distribution corresponding to the associated degrees of freedom and a 95% confidence level for each analyte. The coverage factor is 3.18.

[(b)] The certified value is a weighted mean of the results from two analytical methods, INAA and ICP-OES[61]. The uncertainty listed with each value is an expanded uncertainty about the mean, with the coverage factor, *k* = 2 (approximately 95% confidence interval), calculated by combining a pooled within-method variance with a between-method variance following the ISO/JCGM Guide.

Table S3. Reference mass fraction values for inorganic constituents in $PM_{4.0}$ (SRM2786)

Inorganic compound	Mass Fraction[(a)] (mg/kg)
Antimony	192.1 ± 9.4
Arsenic	36.7 ± 1.1
Calcium	73500 ± 1900
Cesium	4.01 ± 0.22
Chlorine	17390 ± 440
Cobalt	19.55 ± 0.96
Iron	48900 ± 2400
Lanthanum	20.72 ± 0.68
Nickel	243 ± 4[(b)]
Samarium	2.840 ± 0.090
Sodium	14920 ± 370
Titanium	2460 ± 140
Zinc	1793 ± 88

[(a)] Reference values are based on the results from the INAA study unless otherwise noted. The associated uncertainty accounts for both random and systematic effects, but because only one method was used, unrecognized biases may exist for some elements in this matrix. The expanded uncertainty in the reference value is equal to $U = ku_c$, where u_c is the combined standard uncertainty and k is the coverage factor, both calculated according to the ISO/JCGM Guide. The value of u_c is intended to represent, at the level of one standard deviation, the uncertainty in the mass fraction value. Here, u_c accounts for within-method variation and material inhomogeneity. The coverage factor, k, is Student's t-value for a 95% confidence interval with the corresponding degrees of freedom (df = 23).

[(b)] The reference mass fraction value is the mean of the results obtained using one analytical technique (ICP-OES). The expanded uncertainty, U, is calculated as $U = ku_c$, where u_c is the standard error of the dry-mass corrected analyte mean, and the coverage factor, k, is determined from Student's t-distribution corresponding to six degrees of freedom and a 95 % confidence level for each analyte. The coverage factor k is 2.45.

Supplementary reference

S1. DerSimonian, R. & Laird, N. Meta-analysis in clinical trials. *Control Clin Trials*. **7**, 177-188 (1986).
